# Acceptance and affordability of malaria vaccines: issues relating to hesitancy and willingness to pay amongst Nigerian parents of under-five children

**DOI:** 10.1186/s12936-025-05268-y

**Published:** 2025-02-07

**Authors:** Obi Peter Adigwe, Godspower Onavbavba

**Affiliations:** National Institute for Pharmaceutical Research and Development, Abuja, Federal Capital Territory Nigeria

**Keywords:** Vaccination, Immunisation, Immunity, Public Health, Intervention, Payment

## Abstract

**Background:**

With the recent approval of the malaria vaccine by the World Health Organization, it is expected that global acceptance and subsequent uptake of the intervention can help to reduce the burden of the disease in Africa. This study adopted a proactive approach in assessing parents' acceptance of the malaria vaccine, alongside their willingness to pay for the novel public health intervention.

**Methods:**

A national cross-sectional survey was undertaken in Nigeria using a questionnaire as the data collection instrument. The study tool was administered to parents of child-bearing age. Descriptive and inferential statistical analyses were performed using the Statistical Package for Social Sciences (SPSS) software version 25.

**Results:**

A total of 1413 valid responses were received with male (49.5%) and female (50.5%) participants represented by similar proportions. Close to two-thirds (62.5%) of the participants were between the ages of 31 and 40 years, and 47.4% of the participants were educated up to national diploma level. More than two-thirds (69.6%) of the participants indicated that they were worried about side effects that may be associated with the malaria vaccine. A strong majority (90%) of the participants indicated that the vaccine should be administered at no cost to citizens, while 46.7% of the respondents were willing to pay for the malaria vaccination. Levels of education attained by the respondents influenced their willingness to pay for malaria vaccination. This variable also underpinned participants'  reasons for non-acceptance of the vaccine. Those who attained only primary and secondary levels of education were significantly more likely to reject the malaria vaccine because they were against vaccines in general (AOR = 6.63; 95% CI = 1.33 – 39.25; *p* = 0.021).

**Conclusion:**

This study provides critical novel insights which could influence vaccination efforts aimed at reducing the burden of malaria in Nigeria, as well as similar settings.

## Background

Malaria is a life-threatening vector-borne infectious disease. It affects mostly vulnerable groups such as pregnant women and young children living in tropical parts of the world [1]. The disease is spread by female *Anopheles* mosquitoes through transmission of either one or more of the *Plasmodium* species, including *Plasmodium falciparum, Plasmodium ovale, Plasmodium malariae* and *Plasmodium vivax* [2].

Up to 90% of the global incidence and deaths associated with malaria occur in Africa, with the sub-Saharan region accounting for over 70% of the global cases and mortalities [3]. Out of the 93% of the global malaria deaths, under-five children account for 61% in Africa. The disruption caused by the COVID-19 pandemic contributed to the sudden increase of reported cases by more than 14 million incidents and 69,000 deaths compared to the situation in 2019 [4]. Apart from its negative effect on human health, evidence exists associating malaria with significant adverse effects of the global economy, alongside relevant socioeconomic indices [5].

Malaria remains a disease of public health interest and can be controlled through a multi-faceted approach [1]. Over the years, there have been cases of resistant malaria to available medicines for treatment, including artemisinin-based combination therapy [6]. Vaccines, thus, appears to be one important tool that is yet to be fully explored for the control of the disease. Vaccination is the most effective means of preventing infectious disease, as has been illustrated by the eradication of smallpox in humans. Further evidence with other conditions is indicated by remarkable success recorded globally against polio, rabies, and measles [7].

With the development of malaria vaccines, it is envisioned that only universal acceptance and uptake will enable it to provide protection against the condition. If this is achieved the global burden of mortality and morbidity can be reduced significantly. Research had however shown suboptimal childhood vaccination in low and middle-income countries (LMICs). The rate of vaccination coverage among children was as low as 1 in 10 in some parts of Nigeria despite all efforts and strategies employed to reduce low immunization rates, such as the implementation of free vaccine administration for some infectious diseases at public healthcare facilities [8, 9]. The concerns of vaccine acceptance and uptake are further exacerbated by the indications that vaccine financing by development partners in Nigeria may discontinue and pose uncertainties regarding the cost implication for the population [10]. What this means is that the possibility of hesitancy and unwillingness to pay may limit the impact of this new intervention.

Available evidence suggests that several factors such as level of education, concern about safety, and misinformation can influence uptake of a new vaccine [11]. Vaccine campaigns targeting young mothers, especially in societies with poor immunization coverage, may improve vaccine uptake. National Agency for Food and Drug Administration and Control (NAFDAC) has recently given provisional approval for the R21 malaria vaccine for use in Nigeria [12]. A literature search revealed that no study has robustly explored acceptance and willingness to pay for malaria vaccine in Nigeria following the approval. Relevant studies that were undertaken prior to approval of the malaria vaccine were limited to either state or community level. It is against this backdrop that a representative national study was undertaken to assess views of parents of under five children towards malaria vaccination in Nigeria ahead of roll out.

## Methods

### Study design

A national study was undertaken in Nigeria using a cross-sectional design to obtain data from parents of under-five children in the country. A well-structured questionnaire was used for the conduct of the study.

### Study tool

The questionnaire for the study was adapted from previous study on vaccine hesitancy [11]. The questionnaire comprised socio-demographic characteristics and a section assessing views regarding acceptance and payment towards malaria vaccine. The section on vaccine acceptance required the participants to indicate statements that best described the reason they would not want their child to take malaria vaccine. An additional question on acceptance was structured to gain insight into participants’ perception of the benefit and risk of malaria vaccine using a four-point Likert scale which was coded as; 1 = Strongly disagree, 2 = Somewhat disagree, 3 = Somewhat agree, and 4 = Strongly agree. Also, questions on payment for malaria vaccine were structured using the options ‘Yes’ or ‘No’. Furthermore, the maximum cost that the parents were willing to pay for the vaccine was determined using price ranges between ‘less than 500 NGN’ to ‘5000 NGN’.

### Inclusion and exclusion criteria

Inclusion criteria were married adults of child bearing age, and willingness to participate in the study. Non-Nigerians and participants who did not have a child of five years and below were excluded from the study.

### Sampling

The minimum required sample size was calculated to be 1067 participants for a population of approximately 220 million people in Nigeria. This was computed at 95% confidence level, 3% margin of error, and 50% response distribution using the Epi Info software version 7. Participants were recruited using stratified multistage sampling method. Firstly, one state was randomly selected from each of the six geopolitical zones in Nigeria. Three health facilities were randomly selected from each state. In each facility, a number of participants were randomly selected for the study whilst ensuring gender balance. The respondents were made of persons seeking out-patient care for their children who are under five years of age.

### Ethical considerations

Ethical approval was obtained from the National Institute for Pharmaceutical Research and Development Health Research Ethics Committee prior to collection of data. Participation in the study was voluntary as written informed consents were obtained from the participants before questionnaire administration.

### Data collection

Self-completion paper-based questionnaires were administered to the parents of the under-five children at the health facilities they were visited. Completed questionnaires were retrieved from the participants. A total of 300 hard copies of questionnaires were distributed in each of the states making a total of 1800 for the six states. The number was rounded up to this figure so as to ensure enough responses.

### Data analysis

Data analysis was undertaken with the use of Statistical Package for Social Sciences (SPSS) software version 25. Descriptive statistical analysis was undertaken and results presented in percentages and frequencies. Inferential statistical analysis was further carried out to determine association between variables and socio-demographic characteristics. Multinomial logistic regression analysis was used to assess the relationship between participants' reasons for non-acceptance of the malaria vaccine and demographic data. In addition, multivariate binary logistic regression analysis was carried out to test for statistical significance between willingness to pay for malaria vaccination and the sociodemographic characteristics of the participants. Prior to conducting the regression analyses, multicollinearity was checked for and based on the result, some categories in the independent variables were merged. A *p* value of 0.05 or less represented the threshold for statistical significance.

## Results

### Socio-demographic characteristics

Of the 1800 questionnaires that were administered, a total of 1413 copies were completed and returned accounting for a response rate of 78.5%. Male and female participants were of a similar proportion as represented by 49.5% and 50.5% respectively. Respondents between the ages of 31 to 40 years constituted the majority of the study cohort (62.5%), whilst 41 and above represented the least proportion of the sample (9.6%). Close to half of the study participants (47.4%) were educated up to national diploma level. Further details on socio-demographic characteristics are presented in Table 1.

**Table 1:** Socio-demographic characteristics

Variables	Frequency (%)
Gender	
Male	700 (49.5)
Female	713 (50.5)
Age (years)	
≤ 30	393 (27.8)
31–40	884 (62.5)
41 and above	136 (9.6)
Highest educational level	
Primary school	11 (0.8)
Secondary school	275 (19.5)
National Diploma/NCE	669 (47.4)
First degree/HND	400 (28.4)
Postgraduate	53 (3.8)
Occupation	
Unemployed	7 (0.5)
Student	80 (5.7)
Self employed	392 (27.9)
Employed in private sector	701 (49.6)
Employed in government sector	215 (15.3)
Retired	10 (0.7)
Monthly income in NGN	
< 30000	39 (3.8)
30000–59000	396 (28.0)
60000–119000	761 (53.9)
120000–239000	198 (14.0)
240000 and above	11 (0.8)

*NCE* National Certificate of Education, *HND* Higher National Diploma

### Reasons for non-acceptance of malaria vaccine

**Fig. 1:**
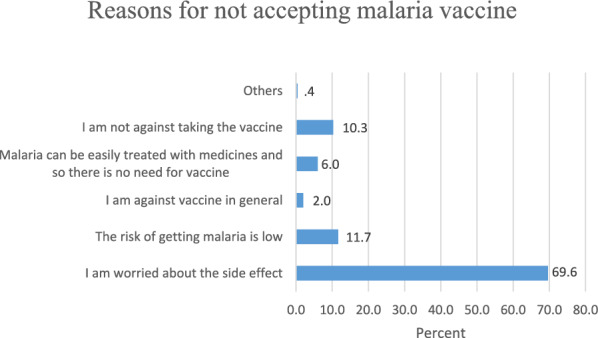
Reasons for not accepting malaria vaccine

Findings presented in Fig. 1 shows that more than two-thirds of the respondents (69.9%) were worried about the potential side effects that may be associated with the newly approved malaria vaccine, and only 10.3% of the respondents indicated that they were not against taking the new intervention.

Despite the high prevalence and endemic nature of malaria in Nigeria and other African countries, 11.7% of the participants indicated that the risk of being infected with malaria was low.

### Benefits of malaria vaccine

**Fig. 2:**
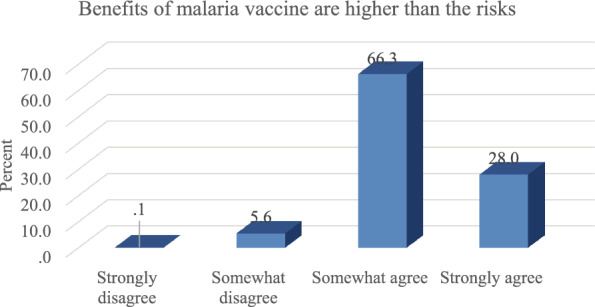
Benefits and risks of vaccine

Vaccines are the most effective tools for preventing the transmission of infectious diseases and before the use of this intervention, there needs to be a clear evidence of its effectiveness. Interestingly, almost all the participants (94.3%) were of the view that the benefits of malaria vaccine were higher than the risks. Further details are presented in Fig. 2.

Although, almost all the participants were in consensus regarding the advantages of malaria vaccine, 5.7% of the study participants were of the view that the risks associated with the intervention outweigh the benefits.

### Willingness to pay for malaria vaccine

Findings from this study revealed that a strong majority of the participants (90%) were of the opinion that malaria vaccine should be administered at no cost to the public, whilst 10% felt otherwise. Also, 42.7% of the respondents showed willingness to pay for malaria vaccine for their children, and 57.2% were unwilling take up the cost for this new intervention.

**Fig. 3:**
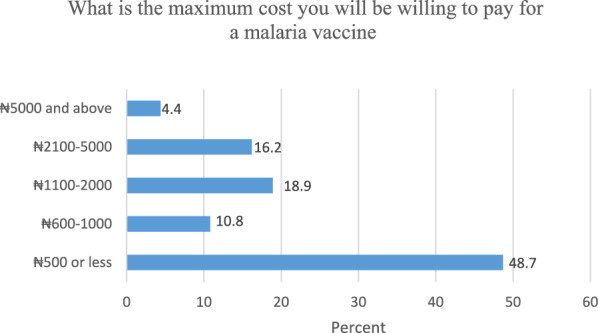
Payment for malaria vaccine

Furthermore, amongst the participants that were willing to pay for malaria vaccination, close to half of the participants (48.7%) indicated that they could afford ₦500 (US$ 0.65) for the vaccine. Further details are presented in Fig. 3.

Findings from this study revealed that only a few of the participants (4.4%) were willing to pay a fee of ₦5000 (US$6.44) and above for their children to be vaccinated against malaria, and 18.9% were willing to provide between ₦1100 and ₦2000.

### Association between socio-demographic characteristics and variables

Multivariable multinomial regression was conducted to determine the relationship between variables and the reasons for non-acceptance of the malaria vaccination by the participants. Results for unadjusted multinomial regression are shown in Table 2. From the findings, participants’ highest level of education and monthly income were associated with reasons for non-acceptance of the vaccine when compared to not being against taking the vaccines.

**Table 2:** Unadjusted multivariable multinomial regression of ‘reasons for non-acceptance of malaria vaccination’ compared to the statement ‘I am not against taking the vaccines’ as reference category

Demography	Reasons for non-acceptance of malaria vaccination							
	I am worried about the side effect		The risk of getting malaria is low		I am against the vaccine in general		Malaria can be easily treated with medicines and so there is no need for vaccine	
	COR (95% CI)	*p*-value	COR (95% CI)	*p*-value	COR (95% CI)	*p*-value	COR (95% CI)	*p*-value
Gender								
Male	+ 1.06 (0.77–1.50)	0.746	+ 1.22 (0.78–1.91)	0.389	+ 1.12 (0.50–2.51)	0.788	+ 1.57 (0.91–2.69)	0.106
Female	Ref		Ref		Ref		Ref	
Age (Years)								
≤ 30	+ 1.20 (0.65–2.21)	0.568	+ 1.87 (0.79–4.41)	0.154	+ 1.98 (0.39–9.98)	0.409	- 0.53 (0.23–1.21)	0.130
31 – 40	+ 1.48 (0.84–2.61)	0.178	+ 1.84 (0.82 –4.14)	0.141	+ 1.62 (0.34–7.70)	0.545	- 0.47 (0.22–1.01)	0.053
41 and above	Ref		Ref		Ref		Ref	
Highest educational qualification								
Primary/Secondary	+ 1.50 (0.89–2.51)	0.125	+ 2.71 (1.42–5.15)	0.002*	+ 6.94 (2.00—24.02)	0.002*	+ 1.84 (0.86–3.96)	0.117
National Diploma/NCE	+ 1.39 (0.95–2.05)	0.092	+ 1.78 (1.06–3.00)	0.030*	+ 3.04 (0.94–9.86)	0.064	+ 1.40 (0.76—2.59)	0.278
First Degree/HND/PG	Ref		Ref		Ref		Ref	
Occupation								
Self employed	+ 1.80 (0.88–3.69)	0.110	− 0.97 (0.41–2.32)	0.947	− 0.97 (0.26–2.63)	0.965	− 0.88 (0.33–2.36)	0.803
Private Sector	+ 1.46 (0.75—2.85)	0.268	− 0.77 (0.34–1.74)	0.529	− 0.36 (0.10–1.36)	0.131	− 0.54 (0.21–1.38)	0.200
Government Sector	+ 1.50 (0.69–3.23)	0.306	− 0.85 (0.33–2.19)	0.740	− 0.55 (0.12–2.58)	0.445	− 0.76 (0.26–2.35)	0.623
Unemployed/Student/Retired	Ref		Ref		Ref		Ref	
Monthly income								
≤ 30000—59000	+ 1.64 (1.00–2.68)	0.048*	+ 1.85 (0.95–3.58)	0.069	+ 5.41 (1.15—25.42)	0.033*	+ 1.87 (0.83–4.19)	0.130
60000—119000	+ 2.07 (1.31–3.26)	0.002*	+ 1.93 (1.04–3.59)	0.037*	+ 3.14 (0.66—14.84)	0.149	+ 1.87 (0.87–4.02)	0.106
120000 and above	Ref		Ref		Ref		Ref	

*COR* Crude Odds Ratio, 95% CI – 95% Confidence interval

The positive ( +) and negative (-) signs before the Odds Ratio are indications of whether the odds increase or decrease respectively. ‘Ref’—reference category. PG – Postgraduate

^*^p < 0.05

From the adjusted multinomial regression presented in Table 3, those who attained primary and secondary levels of education were more likely not to accept the malaria vaccine because they were against the vaccine in general (AOR = 6.63; 95% CI = 1.33–39.25; *p* = 0.021). Another reason was that they felt that the risk of getting malaria is low (AOR = 3.30; 95% CI = 1.45–7.54; *p* = 0.005) and this was also the case of the participants who had National Diploma/NCE (AOR = 1.80; 95% CI = 1.02–3.21; *p* = 0.044). For level of income, those who earned between ₦60,000–₦119,000 were more likely not to accept the malaria vaccine because they were worried about the side effect (AOR = 2.33; 95% CI = 1.35–4.02; *p* = 0.002). Other details are presented in Table 3.

**Table 3:** Adjusted multivariable multinomial regression of ‘reasons for non-acceptance of malaria vaccination’ compared to the statement ‘I am not against taking the vaccines’ as reference category

Demography	Reasons for non-acceptance of malaria vaccination							
	I am worried about the side effect		The risk of getting malaria is low		I am against the vaccine in general		Malaria can be easily treated with medicines and so there is no need for vaccine	
	AOR (95% CI)	*p*-value	AOR (95% CI)	*p*-value	AOR (95% CI)	*p*-value	AOR (95% CI)	*p*-value
Gender								
Male	+ 1.02 (0.71–1.47)	0.908	+ 1.31 (0.71–1.80)	0.601	− 0.96 (0.42–2.20)	0.924	+ 1.60 (0.91–2.81)	0.100
Female	Ref		Ref		Ref		Ref	
Age (Years)								
≤ 30	+ 1.05 (0.54–2.04)	0.880	+ 1.43 (0.57–3.57)	0.442	+ 1.01 (0.18–5.67)	0.988	− 0.36 (0.14–0.90)	0.029*
31 – 40	+ 1.27 (0.69–2.31)	0.444	+ 1.45 (0.62–3.37)	0.390	+ 1.05 (0.21–5.30)	0.946	− 0.36 (0.16–0.82)	0.015*
41 and above	Ref		Ref		Ref		Ref	
Highest educational qualification								
Primary/Secondary	+ 1.48 (0.77–2.83)	0.240	+ 3.30 (1.45–7.54)	0.005*	+ 6.63 (1.33—32.95)	0.021*	+ 1.79 (0.67–4.77)	0.244
National Diploma/NCE	+ 1.29 (0.84–1.97)	0.250	+ 1.80 (1.02–3.21)	0.044*	+ 3.32 (0.89—12.35)	0.073	+ 1.67 (0.83–3.35)	0.149
First Degree/HND/PG	Ref		Ref		Ref		Ref	
Occupation								
Self employed	+ 1.49 (0.71–3.13)	0.298	− 0.85 (0.34–2.11)	0.727	− 0.84 (0.21–3.38)	0.804	− 0.75 (0.27–2.09)	0.576
Private Sector	+ 1.22 (0.56–2.64)	0.619	− 0.96 (0.37–2.58)	0.959	− 0.63 (0.12–3.33)	0.588	− 0.48 (0.16–1.45)	0.194
Government Sector	+ 2.12 (0.84–5.36)	0.112	+ 2.18 (0.67–7.11)	0.196	+ 3.75 (0.45—30.96)	0.220	+ 1.35 (0.35–5.11)	0.664
Unemployed/Student/Retired	Ref		Ref		Ref		Ref	
Monthly Income								
≤ 30000—59000	+ 1.70 (0.90–3.19)	0.101	+ 1.64 (0.70 – 3.83)	0.258	+ 5.55 (0.81—38.34)	0.082	+ 2.31 (0.79 – 6.76)	0.125
60000—119000	+ 2.33 (1.35–4.02)	0.002*	+ 2.20 (1.05 – 4.61)	0.037*	+ 5.18 (0.89—30.10)	0.067	+ 3.17 (1.24 – 8.08)	0.016*
120000 and above	Ref		Ref		Ref		Ref	

*AOR* Adjusted Odds Ratio, 95% CI – 95% Confidence interval

The positive ( +) and negative (−) signs before the Odds Ratio are indications of whether the odds increase or decrease respectively. ‘Ref’—reference category. PG – Postgraduate

^*^p < 0.05

A multivariate binary logistic regression analysis was used to examine factors associated with willingness to pay for malaria vaccination. Table 4 shows the crude odds ratio (COR) and adjusted odds ratio (AOR) for the variables. The odds of male participants being unwilling to pay for the vaccination was 0.99 (AOR) times less than female participants, however, this was not significant (*p* = 0.914). Also, compared to participants who earned ₦120,000 and above, participants who earned between < ₦30,000–₦59,000, as well as those who earned ₦60,000—₦119,000, were more likely to be unwilling to pay for the vaccine (AOR = 1.42 and 1.17 respectively), however, these were not significant. Remarkably, those with primary and secondary schools as their highest level of education were 1.53 times more unwilling to pay for malaria vaccination compared to participants who had higher qualifications (*p* = 0.032). Further details are presented in Table 4.

**Table 4:** A multivariate binary logistic regression analysis of factors associated with willingness to pay for malaria vaccination

Demography	Willingness to pay		COR (95% CI)	*p*-value	AOR (95% CI)	*p*-value
	Yes 593 (42.8)	No 794 (57.2)				
Gender						
Male	296 (42.9)	394 (57.1)	− 0.99 (0.78–1.22)	0.914	− 0.97 (0.78–1.20)	0.745
Female	297 (42.6)	400 (57.4)	Ref		Ref	
Age (years)						
≤ 30	156 (40.2)	232 (59.8)	1.13 (0.76–1.69)	0.548	− 0.96 (0.63–1.47)	0.842
31–40	380 (43.8)	487 (56.2)	− 0.97 (0.67–1.41)	0.889	− 0.85 (0.58–1.25)	0.401
41 and above	57 (43.2)	75 (56.8)	Ref		Ref	
Highest educational level						
Primary/Secondary school	103 (36.9)	176 (63.1)	1.57 (1.16–2.13)	0.004*	1.53 (1.04–2.26)	0.032*
National Diploma/NCE	276 (41.9)	383 (58.1)	1.27 (1.00–1.62)	0.050	1.25 (0.96–1.63)	0.103
First degree/HND/PG	213 (47.9)	232 (52.1)	Ref		Ref	
Occupation						
Self employed	151 (38.8)	238 (61.2)	1.33 (0.84–2.09)	0.220	1.39 (0.87–2.23)	0.167
Employed in private sector	295 (43.1)	390 (56.9)	1.12 (0.72–1.72)	0.623	1.51 (0.92–2.51)	0.105
Employed in government sector	99 (46.7)	113 (53.3)	− 0.96 (0.59–1.57)	0.877	1.61 (0.89–2.91)	0.114
Unemployed/Student/Retired	43 (45.7)	51 (54.3)	Ref		Ref	
Monthly income in naira						
< 30000–59000	165 (38.4)	265 (61.6)	1.55 (1.11–2.16)	0.011	1.42 (0.93—2.18)	0.103
60000–119000	323 (43.4)	422 (56.6)	1.26 (0.92–1.71)	0.147	1.17 (0.81–1.68)	0.397
120000 and above	101 (49.0)	105 (51.0)	Ref		Ref	

*COR* Crude Odds Ratio, *AOR* Adjusted odds ratio, 95% CI – 95% Confidence interval. The positive ( +) and negative (−) signs before the Odds Ratio are indications of whether the odds increase or decrease respectively

^*^p < 0.05

## Discussion

This national study has revealed novel insights regarding acceptance and willingness to pay for malaria vaccination in Nigeria. Findings from this study suggest that majority of the participants expressed some level of hesitancy towards the newly approved malaria vaccines. Their major concern was the side effects that may be associated with the intervention, and this was similar to the fears expressed towards COVID-19 vaccine [13, 11]. In the Nigerian setting, adverse effects associated with vaccines have previously been identified as a significant barrier to childhood immunization coverage [8]. The RTS, S malaria vaccine was approved by the World Health Organization (WHO) on October 6, 2021 and prior to this time, relevant studies that were undertaken in African setting with respect to this public health tool showed high level of acceptance amongst parents [4]. In Nigeria, the R21 malaria vaccine was provisionally approved for use in the country in April 2023. This makes Nigeria the second country to grant such approval in the world after Ghana [14]. Vaccine hesitancy was reignited following the COVID-19 pandemic. Several negative campaigns targeted at discrediting vaccines during the hit of COVID-19 may have contributed towards the current high rate of hesitancy expressed by participants in this study [15].

A failure to address the concerns expressed by respondents in this study could delay or prevent the achievement of optimal vaccination coverage alongside other possible public health consequences. Given the high prevalence and mortality associated with malaria, especially among children under five years of age, it is important that contextual strategies be adopted to reduce malaria vaccine hesitancy in Nigeria and promote uptake [16]. Vaccine hesitancy is not new, the phenomenon has been in existence before the emergence of COVID-19 [17]. There is a need to therefore explore novel strategies to deal with misconceptions towards the use of vaccines. In addition to safety, efficacy can also play critical role in improving acceptance following the introduction of a new vaccine.

Interestingly, almost all the participants were of the view that the benefits of malaria vaccine outweigh the risks, suggesting that they were aware of the role and importance of vaccination in reducing the burden of disease. Historically, significant empirical evidence exists, establishing the general safety and effectiveness of vaccines [18]. Even in specific population groups such as children, adolescents, and adults, the findings from the extant literature support the safety of vaccination as a public health intervention [19]. Although there is evidence that adverse effects may arise from vaccination, they are generally mild [20], especially when viewed in relation to the public health benefits. The need for continuous enlightenment of the public about vaccines’ safety cannot be overemphasized. Targeted campaigns that highlight simple risk–benefit analysis of the intervention can also help improve awareness and consequent acceptance.

In this study, a strong majority of the participants indicated that malaria vaccines be administered at no cost, and less than half of the study participants were willing to pay a fee for their children to be vaccinated against malaria. These findings suggest that attributing a fee to malaria vaccine in Nigeria will reduce uptake and increase hesitancy amongst the public. This can consequently delay the elimination of malaria in this setting. Previous studies had demonstrated that the rate of acceptance of vaccines is higher when it is made free to the public [21–23].

The inferential statistical analysis undertaken revealed significant relationship between socio-demographic characteristics and reasons for non-acceptance of malaria vaccination. Specifically, level of education and monthly income were attributed to the reasons for the non-acceptance of the vaccine and this is in line with the studies conducted in Hungary [24], Croatia [25], Poland [26] and Nigeria [27]. Higher educational attainment is generally linked to increased health literacy, which positively influences vaccine acceptance. Additionally, lower income levels may correlate with reduced access to healthcare resources and information, further exacerbating fears regarding vaccination. These findings are indications that targeted interventions to improve acceptance can begin through the educational sector. Also, a general evaluation and improvement of salary structure for lower-income earners should be considered for more favourable access to appropriate information that promotes vaccine uptake.

Furthermore, the participants’ level of education was observed to have influence on willingness to pay. Participants with higher level of education were more likely to pay for the intervention, and this was in tandem with previous findings in Ethiopia [28] and Nigeria [29]. The possible reason for this could be attributed to the widespread conspiracy that followed the development of COVID-19 vaccines. The misinformation that was circulated during COVID-19 may have contributed to this finding.

To achieve the WHO’s target to reduce global malaria incidence and mortality rates by at least 90% by the year 2030 [30], increasing the uptake of vaccines against malaria is an important strategy that can considered in the attainment of this critical milestone. Within this context, vaccination against malaria is an important tool for reducing the burden of the disease in Nigeria and other African countries. The widespread adoption and uptake of vaccination against malaria can significantly contribute to a rapid reduction of the high burden of the disease in the African setting. Emergent findings advocating the improvement of community engagement and provision of relevant information about safety and benefits are critical for increasing acceptance of malaria vaccines. Given the health and socioeconomic impact associated with the high burden of malaria, the novel findings from this study can underpin policies that significantly reduce related morbidity and mortality indices.

## Strength and limitation

This study did not include illiterates, as questionnaires were only distributed to persons that were able to read and complete them. This potentially limits the study, since evidence from this demography remains important for decision making. This limitation was however mitigated by targeted efforts at achieving a representative sampling through  the study's large sample size and the use of multistage sampling technique. Further studies can adopt mixed methods which include interviews or focus group discussions where this demography can actively participate.

## Conclusion

This study provides novel insights on malaria vaccination and willingness to pay amongst parents of under five children in Nigeria, and concerns about malaria vaccines were identified. Understanding the factors that influence people's attitudes towards accepting or rejecting vaccines for themselves or their children can help improve uptake and reduce hesitancy. To avoid poor implementation outcomes associated with vaccine hesitancy, there is a need to comprehensively address population’s concerns about safety through enlightenment campaigns. Findings from this study revealed that paying a fee for malaria vaccination may reduce the uptake of the vaccine. As well as the cost considerations, policies prioritizing the adoption of incentives may increase acceptance, especially in rural communities. Policymakers in Government and other stakeholders in the healthcare sector can leverage these emergent findings to develop contextual strategies aimed at achieving optimal immunization coverage.

The new evidence revealed by this study can contextualize healthcare sector capacity building and consequently make the intervention more effective. Healthcare practitioners’ misconceptions concerning vaccines can interfere with their ability to effectively communicate the benefits of vaccination to their patients as well as the general public. Relevant training programmes that address safety and efficacy of vaccines, as well as other critical immunization topics will not only build practitioner confidence it will also improve capacity to communicate vaccination benefits. Given the national and global significance of this issue, further studies are recommended to enable a more robust and comprehensive exploration. Future findings that build on the foundation of this study will be invaluable in developing contextual strategies that address hesitancy and various population concerns, not just for malaria but for other lifesaving vaccines as well.

## Data Availability

The datasets used and/or analysed during the current study are available from the corresponding author on reasonable request.

## Abbreviations

SPSS: Statistical package for social sciences
LMICs: Low and middle-income countries
NAFDAC: National agency for food and drug administration and control
NCE: National certificate of education
HND: Higher national diploma.
WHO: World health organization
